# Genome of *Spea multiplicata*, a Rapidly Developing, Phenotypically Plastic, and Desert-Adapted Spadefoot Toad

**DOI:** 10.1534/g3.119.400705

**Published:** 2019-10-02

**Authors:** Fabian Seidl, Nicholas A. Levis, Rachel Schell, David W. Pfennig, Karin S. Pfennig, Ian M. Ehrenreich

**Affiliations:** * Molecular and Computational Biology Section, Department of Biological Sciences, University of Southern California, Los Angeles, CA 90089, and; † Department of Biology, University of North Carolina, Chapel Hill, NC 27599

**Keywords:** spadefoot toad, Spea, phenotypic plasticity, hybridization, rapid development, genome, gene expression

## Abstract

Frogs and toads (anurans) are widely used to study many biological processes. Yet, few anuran genomes have been sequenced, limiting research on these organisms. Here, we produce a draft genome for the Mexican spadefoot toad, *Spea multiplicata*, which is a member of an unsequenced anuran clade. Atypically for amphibians, spadefoots inhabit deserts. Consequently, they possess many unique adaptations, including rapid growth and development, prolonged dormancy, phenotypic (developmental) plasticity, and adaptive, interspecies hybridization. We assembled and annotated a 1.07 Gb *Sp. multiplicata* genome containing 19,639 genes. By comparing this sequence to other available anuran genomes, we found gene amplifications in the gene families of *nodal, hyas3, and zp3* in spadefoots, and obtained evidence that anuran genome size differences are partially driven by variability in intergenic DNA content. We also used the genome to identify genes experiencing positive selection and to study gene expression levels in spadefoot hybrids relative to their pure-species parents. Completion of the *Sp. multiplicata* genome advances efforts to determine the genetic bases of spadefoots’ unique adaptations and enhances comparative genomic research in anurans.

With at least 7,040 species (AmphibiaWeb 2018), frogs and toads (anurans) occur across diverse habitats and exhibit a stunning array of adaptations (Duellman and Trueb 1986; Halliday 2016). Moreover, anurans are critical, but increasingly threatened, components of most ecosystems and thus serve as key bioindicators (Stuart *et al.* 2004). Despite their importance to fields from developmental biology and physiology to ecology and evolution, genomic resources are relatively scarce for anurans. Indeed, fewer genomes are available for anurans than for most other major groups of vertebrates, with only seven anurans sequenced: the Western clawed frog, *Xenopus tropicalis*, and the closely related African clawed frog, *Xenopus laevis* (Hellsten *et al.* 2010), the Tibetan Plateau frog, *Nanorana parkeri* (Sun *et al.* 2015), the American bullfrog, *Rana* (*Lithobates*) *catesbeiana* (Hammond *et al.* 2017), the Cane toad, *Rhinella marina* (Edwards *et al.* 2018), the Strawberry Poison frog (*Oophaga pumilio*) (Rogers *et al.* 2018), and the African bullfrog (*Pyxicephalus adspersus*) (Denton *et al.* 2018). This paucity of genomes limits the use of anurans as model systems for many important biological questions, especially given their deep levels of divergence (Bossuyt and Roelants 2009).

Here, we present a draft genome of a New World spadefoot toad, the Mexican spadefoot toad, *Spea multiplicata* (family Scaphiopoididae; Figure 1A,B). New World spadefoot toads (hereafter, ‘spadefoots’) comprise seven diploid species, two of which––*Scaphiopus holbrookii* and *Sc. hurterii*––occur in relatively mesic eastern and central North America, and five of which––*Sp. multiplicata*, *Sp. bombifrons*, *Sp. hammondii*, *Sp. intermontana*, and *Sc. couchii*––inhabit xeric western North America. Crucially, relative to the other frogs and toads with published genomes, spadefoots fill an unsequenced gap of > 200 My on the anuran phylogeny (Figure 1A). Additionally, spadefoots possess some of the smallest anuran genomes of between 1.0 and 1.4 Gb (Gregory 2018). By contrast, the genomes of other sequenced diploid anurans range from 1.7 Gb for *X. tropicalis* (estimated via karyotype) (Hellsten *et al.* 2010) to a 5.8 Gb assembly size for *R. catesbeiana* (Hammond *et al.* 2017).

**Figure 1:**
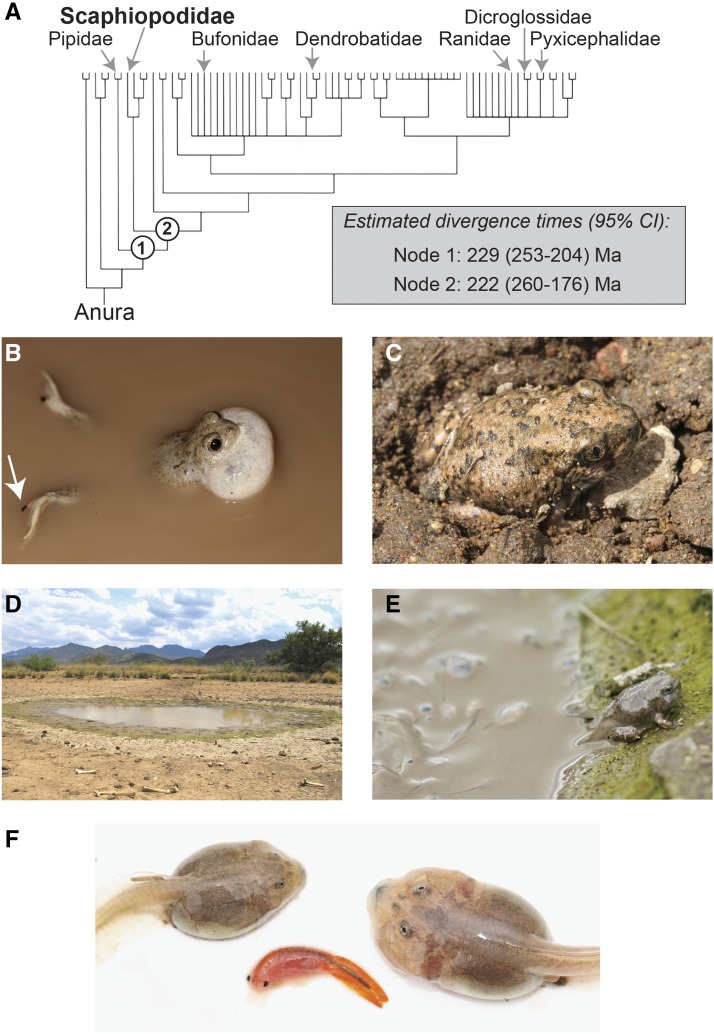
Evolution and natural history of New World spadefoot toads. A Phylogenetic relationships among the New World spadefoot toads (genera *Spea* and *Scaphiopus*; family Scaphiopodidae) and six other species of sequenced anurans: *Xenopus laevis* (Pipidae), *Rhinella marina* (Bufonidae), *Oophaga pumilio* (Dendrobatidae), *Rana* (*Lithobates*) *catesbeiana* (Ranidae), *Nanorana parkeri* (Dicroglossidae), and *Pyxicephalus adspersus* (Pyxicephalidae) [phylogeny after AmphibiaWeb (2018); estimated divergence times from Bossuyt and Roelants (2009)]. B Mexican spadefoot toads, *Spea multiplicata*, possess numerous adaptations for dealing with desert conditions including a keratinized spade on their hind feet (arrow), which enables them to C burrow underground. D They emerge for only a few weeks each year to feed and to breed in temporary, rain-filled pools. e Spadefoot tadpoles exhibit rapid, and adaptively flexible, larval development (here, a metamorph emerges from a drying pond). F They also produce alternative, environmentally induced morphs: a slower developing omnivore morph (left) and a more rapidly developing carnivore morph (right), which is induced by, and specializes on, animal prey, such as fairy shrimp (center).

Spadefoots serve as important models in ecology and evolution, owing to their unusual ecology, rapid development, and striking phenotypic plasticity. For example, spadefoots cope with their arid habitat by burrowing underground (Ruibal *et al.* 1969) and estivating for a year or longer (Mayhew 1965; Mcclanahan 1967; Seymour 1973), emerging for only a few weeks following warm rains to feed and breed in short-lived pools (Figure 1C, D) (Bragg 1965). Although these highly ephemeral pools are inaccessible to most anurans, spadefoot tadpoles can survive in them by developing rapidly––in some cases metamorphosing in eight days post-hatching (Figure 1E) (Newman 1989). Spadefoots also exhibit multiple forms of phenotypic plasticity that further hastens their development and allows them to thrive in environments (such as deserts) where rainfall is highly variable (Tinsley and Tocque 1995). Specifically, spadefoot tadpoles can facultatively speed up (Denver *et al.* 1998; Morey and Reznick 2000; Boorse and Denver 2003; Gomez-Mestre and Buchholz 2006) or slow down (Newman 1992; Denver *et al.* 1998; Morey and Reznick 2000) development in response to the environment. Additionally, whereas most anuran tadpoles are omnivores and exhibit traits adapted for feeding on detritus and plankton (Wells 2007), *Spea* tadpoles can develop into an alternative––and more rapidly developing––‘carnivore’ ecomorph, which exhibits enlarged jaw muscles and mouthparts for capturing and consuming large animal prey (Figure 1F) (Pfennig 1990; Pfennig 1992a; Pfennig 1992b; Levis *et al.* 2015; Levis *et al.* 2018). This carnivore morph has an additional advantage in a rapidly drying pond: it can reduce competition and further enhance growth and development by eating other tadpoles (Pfennig 2000). Finally, as adults, when breeding in shallow, rapidly drying ponds, *Sp. bombifrons* females preferentially mate with sympatric *Sp. multiplicata* males, thereby producing hybrid tadpoles that develop even faster than pure-species tadpoles (Pfennig 2007). Genome resources for spadefoots will greatly enable further work on understanding spadefoot’s unique characteristics.

In this paper, we performed a combination of long- and short-read sequencing on Mexican spadefoots, *Sp. multiplicata*, and produce a draft genome for this species. By comparing this genome to other available anuran genomes, we identify several distinctive gene amplifications, as well as factors contributing to the substantial genome size variation found among anurans. We then leverage the *Sp. multiplicata* genome as a platform for exploring evolution in two ways. First, we produce short-read, whole genome sequencing data for three other species of spadefoots: Plains spadefoots, *Sp. bombifrons*, Couch’s spadefoots, *Sc. couchii*, and Eastern spadefoots, *Sc. holbrookii*. We obtain thousands of protein-coding gene sequences for these species by mapping the data against *Sp. multiplicata* gene models. This allows us to identify genes exhibiting different selection pressures in *Spea* or *Scaphiopus*, including positive selection, thereby providing insights into specific genes that may underlie adaptive evolution in these genera. Second, we generate transcriptome data for *Sp. multiplicata* and *Sp. bombifrons* tadpoles, as well as for tadpoles produced by hybridizing these species, thereby providing insights into why hybridization might be ecologically and evolutionarily significant in *Spea* (Pfennig 2007; Pierce *et al.* 2017).

Together, our results demonstrate how the *Sp. multiplicata* genome can facilitate genetics and genomics research in spadefoots. Ultimately, such research promises to provide key insights into the distinctive phenotypes of these unique organisms.

## Materials and Methods

### Genome assembly

We selected an adult male *Sp. multiplicata* that had been collected in July 2011 at a breeding aggregation in an ephemeral pond (‘410 Pond’) 20 km SSE of Portal, Arizona USA (31.7384, -109.1). Immediately after euthanizing the male, we removed and homogenized his liver and extracted from it high molecular weight DNA using Qiagen 500G Genomic-tip columns. Additional tissue from this specimen was stored at the North Carolina Museum of Natural Sciences under the identifier NCSM84230. Three types of whole genome sequencing data were generated: Illumina, PacBio, and Oxford Nanopore. Illumina sequencing libraries were constructed with the Illumina Nextera kit. Five replicate Illumina sequencing libraries were prepared, multiplexed using barcoded adapters, and then sequenced at the USC Molecular Genomics Core on an Illumina NextSeq using the 150 bp paired-end kit. PacBio libraries were generated and sequenced on a Pacbio Sequel by the UC Irvine Genomics High-Throughput facility. We also generated Oxford Nanopore long-read libraries, which we sequenced on two 2D DNA chips using a Mk1b Oxford Nanopore Minion. More information about the sequencing data are provided in Supplementary Table 1.

Assembly was performed using all long- and short-read sequencing data. After trying multiple assemblers, we found that MaSuRCA v3.2.1 (Zimin *et al.* 2013) produced the most contiguous assembly. Because MaSuRCA uses Quorum to perform error correction internally, we inputted raw read data into the program. We employed a kmer size of 51, a cgwError rate of 0.15, and a jellyfish hash size of 6x10^10^.

Following completion of the assembly, duplicate contigs were identified using the LAST aligner (Kielbasa *et al.* 2011) with default parameters. All contigs were mapped against all other contigs. If a contig was entirely contained within another contig, with the overlapping regions showing similarity ≥ 0.9, we classified the smaller contig as a duplicate. Such duplicates were removed from the assembly and excluded from all subsequent analyses. We termed this filtered assembly our “contig assembly” and used it in for downstream comparisons to *X. tropicalis*. Characteristics of the contig assembly are described in row three of Supplementary Table 2.

We next ran RepeatModeler v1.0.4 (Smit *et al.* 2013-2015) on the assembly, using default settings. Repetitive elements identified in the *Sp. multiplicata* genome were combined with RepDB volume 16, issue 12 (Bao *et al.* 2015). RepeatMasker v4.0.7 (Smit *et al.* 2013-2015) with -e ncbi was then used to mask repetitive DNA. Characteristics of the repetitive DNA identified in the assembly are described in Supplementary Table 3.

Anuran genomes have a slow rate of structural evolution and high structural similarity has been shown between *N. parkeri* and *X. tropicalis* (Sun *et al.* 2015). In addition, *Spea multiplicata* has a similar number of chromosomes to *X. tropicalis* (n = 13 and n = 12) (Wasserman 1970; Hellsten *et al.* 2010). Thus, we attempted to scaffold the repeat-masked *Sp. multiplicata* contigs using the *X. tropicalis* genome as a reference. This was done in Chromosomer v0.1.4 (Tamazian *et al.* 2016) with a gap length setting of 100 bases.

To assess the quality of the scaffolded assembly, we applied BUSCO v2.0 (Simão *et al.* 2015) to both repeat-masked contigs and scaffolds. BUSCO performs BLASTs against an assembly to ascertain the presence or absence of proteins known to be highly conserved among all members of a phylogenetic branch. Assembly completeness was assessed based on the metazoan gene set, as this set has been previously used in other published assembly reports (Hammond *et al.* 2017). The scaffolds showed significantly improved contiguity relative to the contigs (row four of Supplementary Table 2), indicating that scaffolding improved the assembly.

Lastly, we unmasked repeats in the scaffolds. This scaffolded assembly without repeat masking is what we refer to throughout the paper as the *Sp. multiplicata* ‘assembly.’

### Annotation of protein-coding genes

We performed *ab initio* protein-coding gene prediction using Augustus v3.2.3 (Stanke and Morgenstern 2005; Stanke *et al.* 2008). However, to first generate a training set for Augustus, we empirically annotated a subset of genes in *Sp. multiplicata*. To do this, we utilized previously generated RNA-seq data from tadpoles (Seidl *et al.* 2019). RNA-seq reads were mapped to the assembly using Tophat2 v2.1.1 (Kim *et al.* 2013). We then extracted the nucleotide sequences of parts of the assembly covered by the RNA-seq data. Best matches for these sequences in *X. tropicalis* were obtained by comparing six-frame translations of the *Sp. multiplicata* data against *X. tropicalis* v9.1 peptides obtained from Xenbase (Karimi *et al.* 2018) using Blastx v2.2.30 (Camacho *et al.* 2009). Putative translation start sites were defined as the ATG in the *Sp. multiplicata* sequence closest to the beginning of the alignment. This resulted in a set of 1,478 empirically defined gene models for which the *Sp. multiplicata* peptide spanned ≥ 80% of the *X. tropicalis* match with ≥ 30% sequence identity and a putative translation start site was found.

To train Augustus, we randomly split our empirically defined gene set into a training set (1,000 genes) and a verification set (478 genes). We then ran the Augustus etraining pipeline to estimate parameters that accurately described features of protein-coding genes in *Sp. multiplicata*. We further optimized the parameter estimates using the optimize_augustus.pl script.

After estimating species-specific parameters, we ran Augustus to annotate protein-coding genes in the contig assembly, as well as in the scaffolded assembly. We performed gene prediction in both assemblies to determine the effectiveness of scaffolding against *X. tropicalis*. Augustus produced a total of 81,079 genes from our contigs and 42,671 genes from our scaffolds (Supplementary Data 1; Supplementary Figure 3). The average gene size of the two sets was 316.9 and 365.8 peptides respectively. We took this as evidence that scaffolding enabled better gene prediction than the contigs alone. We compared the protein sequences predicted by Augustus against the *X. tropicalis* (Karimi *et al.* 2018) database using global_search in Usearch v10.0.240 (Edgar 2010). To obtain a high confidence set of 19,639 protein-coding genes, we filtered the complete set of annotated protein-coding genes using multiple criteria. We required proteins to be 30 amino acids or larger (for comparison, the smallest protein in *X. tropicalis* is 33 amino acids long). Also, relative to their best *X. tropicalis* match, we employed thresholds of ≥30% identity, ≥30% target coverage, and ≥75% query coverage.

### Gene ontology analysis

We obtained full gene sets for all publicly available genomes with annotations, namely *X. tropicalis*, *R. marina*, *N. parkeri*, and *R. catesbeiana*, from Xenbase (Karimi *et al.* 2018) or NCBI. We assigned each gene in each species a uniprot ID (Uniprot Consortium 2019) by determining its best match in a SWISS-prot database, retrieved from Uniprot, including anurans, human, and zebrafish (Supplementary Data 4). To generate these matches, we performed VSEARCH (Rognes *et al.* 2016) global pairwise alignment, returning only the best match alignment showing ≥30% identity. We then assigned Biological Process gene ontology (GO) terms to each gene based on its best match uniprot ID.

To determine if *Sp. multiplicata* showed enrichment or depletion of any GO term relative to the other species, we performed chi-square tests for each term (Cai *et al.* 2006). Specifically, we counted the number of genes with and without a given term in each species and compared these values using a series of pairwise chi-square tests. We then corrected for multiple testing using FDR (Benjamini and Hochberg 1995) implemented with the qvalue function in R (Bass *et al.* 2018). A GO term was considered significant if all its pairwise chi-square test results were significant at an FDR below 0.05 and if *Sp. multiplicata* had more (or fewer) genes with that term than each of the other species. If *Sp. multiplicata* was not significantly enriched or depleted for a given GO term relative to every other species, we did not consider the GO term significant in our overall analysis.

### Copy number analysis

We compared the peptide sequences of our gene models, as well as the peptide models of other published anuran assemblies and annotations against the vertebrate SWISS-prot (Bairoch and Apweiler 2000) database. To decrease the likelihood of false positives, we first removed all genes with at least ≥30% identity and ≥90% target coverage. We then counted the number of matches in each species for each of the 18,341 genes in the database. We compared the counts across all five anurans and looked for cases of enrichment specific to *Spea* (Supplementary Table 6). For the purposes of this paper, we defined a gene as enriched specifically in *Sp. multiplicata* if it was present in at least twice the number of copies in *Sp. multiplicata* compared to all the other sequenced anurans, with *Sp. multiplicata* having at least five copies.

To supplement the five anuran sequences we retrieved peptide sequences of *nodal*, *hyas*, and *zp3* from human and zebrafish from uniprot (Uniprot Consortium 2019). We aligned the peptide sequences of all seven species using Muscle v3.8.31 (Edgar 2004) and removed all positions where any species had a gap. We used the R package phangorn (Schliep *et al.* 2017) to calculate maximum likelihood models of amino acid substitution distance using optim.pml with model = WAG and stochastic rearrangement. To further investigate the *nodal* expansion, we included matches to all *nodal* related genes in the SWISSprot database and reduced our coverage requirement to ≥50% for all five anuran species (Supplementary Table 6),

We used short-read sequencing data to verify gene amplifications detected in the assembly. We mapped short-read data from *Sp. multiplicata* against the gene alignments generated for our PAML analyses using bwa v0.7.12 (Li and Durbin 2009) and extracted per base coverage information using the bedtools (Quinlan and Hall 2010) genomecov module. We then divided the median coverage values of our enriched gene models by the median coverage across all gene models to generate a fold coverage measurement. To estimate the total number of gene models in each family we summed fold coverage.

### dN/dS analysis

We sampled, preserved, and stored at the North Carolina Museum of Natural Sciences a single adult from *Sp. bombifrons* (NCSM84228), *Sc. holbrookii* (NCSM84231), and *Sc. couchii* (NCSM84229). We used Qiagen 500G Genomic-tip columns to extract DNA from liver tissue. For each sample, we constructed between three and five replicate Illumina Nextera libraries using the same DNA but different tagmentation and PCR steps following standard protocols. Indexed libraries were then sequenced on an Illumina NextSeq using the 150 bp paired-end kit at the USC Molecular Genomics Core.

We performed traditional short-read assembly on the data from *Sp. bombifrons*, *Sc. couchii*, and *Sc. holbrookii*. We trimmed short reads to remove low-quality bases using Trimmomatic (Bolger *et al.* 2014) and then used the trimmed reads as inputs for the SOAPdenovo2 v2.04 assembler (Luo *et al.* 2012), and ran the assembler across a range of kmers (31, 41, 51, 61, 71, 81, 91, 101) for each species. A single contig set was selected for further use based on the assembly quality parameters, with an emphasis on a total assembly size of ∼1 Gb (Supplementary Table 10). We aligned the contigs of these assemblies to the nucleotide sequences of our predicted genic models using lastal (Kielbasa *et al.* 2011) with default settings. The contig with the best match score for each exon from each species was used to generate an alignment for each gene. Bases that were not covered by the top match contig were encoded as N. We were able to generate alignments for 25,382 genes with partial data from each species.

We used Phylogentic Analysis by Maximum Likelihood (PAML) v4.9 (Yang 2007) to estimate dN/dS (ω) for all genes alignments. We used the cleandata = 1 option to remove all sites where any species had 1 or more Ns in the codon. We utilized the same two models on two distinct mid-point rooted tree topologies. In the first model, a single ω was estimated across all branches. In the second model, two ω parameters were estimated, one for *Spea* (*Sp. multiplicata*, *Sp. bombifrons*) and one for *Scaphiopus* (*Sc. couchii*, *Sc. holbrookii*). We removed those genes with any ω estimates of 999 as well as those with fewer than 50 sites. We then further removed all genes without ≥ 80% of the gene model covered in all four species. This left a total of 1,967 genes. Significance of two ω models relative to one ω models was determined using likelihood ratio tests, with FDRs estimated as above (Bass *et al.* 2018). Typically, ω > 1 indicates positive selection, ω < 1 indicates purifying selection, and ω = 1 indicates neutral evolution. However, here, hypothesis tests were performed by comparing ω between *Spea* and *Scaphiopus*, rather than by comparing ω in a given species against a model where ω = 1 (Yang 2007). To account for this, we used a more stringent threshold for positive selection (ω ≥ 2). Likewise, we also used a more stringent threshold for calling genes as experiencing purifying selection (ω ≤ 0.5).

We performed GO enrichment tests on genes operationally defined as experiencing positive selection in *Spea*. To do this, we compared the number of genes with or without a given GO term in the set of genes under positive selection in *Spea* to the number of genes with or without a given GO term in those genes not under positive selection in *Spea*. For each GO term, a chi-square test was performed using the chisq.test function in R and multiple testing correction was conducted on the entire set of tests in qvalue (Bass *et al.* 2018). We then manually explored information regarding the parent-child relationships of each term, the full description of the GO term, and terms with which a focal term frequently co-occurs using the QuickGO online resource (Binns *et al.* 2009) to determine potential biological relevance (‘Functional grouping’ in Supplementary Table 9). Note that, unlike other analyses in the paper, terms reported as significant in this analysis were identified at an FDR threshold of 0.065.

### Analysis of gene expression in pure species and their hybrids

We contrasted gene expression in tadpoles between the *Spea* species and their hybrids as follows. To generate pure-species tadpoles, we bred pairs of *Sp. multiplicata* adults and pairs of *Sp. bombifrons*. To create hybrid tadpoles, we paired *Sp. multiplicata* adults with *Sp. bombifrons* adults. We generated hybrids of both maternal cross directions. All adults were wild-caught and maintained in lab facilities. To induce breeding, we injected adults with 0.07 mL 0.01ug/ml gonadotropin releasing hormone (GnRH) agonist. Males and females were placed as pairs in separate aquaria with 10 L of dechlorinated water and allowed to oviposit. We generated at least three replicate families per cross type. After egg release was complete, adults were removed and the eggs were aerated until they hatched. When tadpoles were swimming freely, we selected 16 tadpoles at random from each family; divided these tadpoles into two groups of eight; and placed each group in a tank (34 cm X 21 cm X 11.5 cm) filled with dechlorinated water. All tadpoles were fed their natural diet of shrimp and detritus *ad libitum*. On day 10 after fertilization (comparable to stage 47 in *X. tropicalis*), we killed tadpoles by placing them in MS-222 and then immediately froze them in liquid nitrogen. Thus, all tadpoles were the same age, but not necessarily the same developmental stage, at sampling.

We sampled seven tadpoles each of pure *Sp. multiplicata*, pure *Sp. bombifrons*, and 6 and 8 tadpoles respectively for each type of interspecies hybrid combinations (*i.e.*, female *Sp. multiplicata* x male *Sp. bombifrons* and female *Sp. bombifrons* x male *Sp. multiplicata*). We extracted RNA from whole tadpoles using a combination of TRIzol Reagent and the Ambion PureLink RNA Mini Kit according to Levis *et al.* (2017) and submitted the samples to Cornell Capillary DNA Analysis for preparation and sequencing of 3′ RNA-seq libraries. We trimmed the resulting short reads for poly-A tails and adapter contamination using Trimmomatic (Bolger *et al.* 2014), and aligned individual libraries, as well as pooled libraries, to our scaffolds using STAR aligner(Dobin *et al.* 2013).

3′ RNA-seq only generates reads from the 3′ ends of transcripts, resulting in peaks of data (Beck *et al.* 2010). To identify transcription peaks, we selected the base with maximum coverge for each region with continuous coverage above 50. We then extracted coverage from each individual sample for each of these peaks. We normalized by library size (millions of reads) and log2 transformed the resulting values before performing digital normalization across all data. We calculated the mean-fold coverage for all peaks for pure individuals and hybrids. Gene-specific ANOVA models were then used to identify genes showing significant differential expression between the species. For each gene, we fit the following model:

expression=species+error,

where *expression* corresponds to vector of log_2_ expression measurements for the samples, *species* is a vector containing the species from which each measurement was taken, and *error* denotes the vector of residuals. These models were fit only to data from the pure species, using the aov function in R. P-values for the models were obtained and corrected for multiple testing using ‘qvalue’ (Bass *et al.* 2018), with a significance threshold of FDR ≤ 0.05. After identifying these differentially expressed genes in pure species, we then examined their log2 expression levels in hybrids. Mean expression levels for a given gene within a particular sample class were determined by computing the arithmetic mean of all measurements for that gene in the appropriate class using the mean function in R.

### Data availability

The genome, annotation, and raw data are available through NCBI under the BioProject identifier PRJNA529692. Supplemental material available at figshare: https://doi.org/10.25387/g3.8303672.

## Results

### Properties of the Sp. multiplicata genome

We generated a *Sp. multiplicata* draft genome using a combination of high-coverage long- and short-read sequencing (Supplementary Table 1), hybrid read assembly, and scaffolding of contigs against the *X. tropicalis* reference genome (Supplementary Table 2; Methods). We obtained 84,984 contigs summing to a total haploid genome size of 1.09 Gb with an N50 of 29,771 bp and a maximum contig length of 401,788 bp. Our scaffolded assembly of ∼1.07 Gb, is consistent with historical, densitometry-based genome-size estimates (Sexsmith 1968; Bachmann 1972). The draft genome consisted of 49,736 scaffolds with a scaffold N50 of 70,967 bp and a maximum scaffold size of 60,197,306 Mb. This assembly contiguity is similar to other recently published genomes (Supplementary Table 3). Thirty-two percent of the assembly was comprised of repetitive DNA, with 18%, 5%, 3.9%, 2.5%, and 2.4% of the genome annotated as unclassified interspersed repeats, LINEs, transposable DNA elements, long terminal repeats, and simple repeats, respectively (Supplementary Table 4). We used Benchmarking Universal Single-Copy Orthologs (BUSCO) (Simão *et al.* 2015) to check draft genome completeness (Methods). Among the 978 genes in BUSCO’s metazoan database, 878 (89.8%) were complete, whereas 47 (4.8%) were incomplete and 53 (5.4%) were absent, which is similar to the other recently published anuran genomes (Sun *et al.* 2015; Hammond *et al.* 2017; Edwards *et al.* 2018).

After confirming a high level of genome completeness, we used the software package AUGUSTUS (Stanke and Morgenstern 2005) to perform *ab initio* prediction of protein-coding genes (Supplementary Data 1; Methods). We then BLASTed the predicted proteins against the proteome of *X. tropicalis* and filtered them using multiple quality-control criteria (Methods). Comparisons of the proteome of *Sp. multiplicata* to those of the other five anurans suggests our approach yielded high quality models (Supplementary Figure 1). We thereby identified 19,639 protein-coding gene models, which were on average 1,370 bp excluding introns and 9,398 bp including introns (Supplementary Data 1). This number of genes in *Sp. multiplicata* is slightly lower than, but comparable to, the range of gene numbers reported for the four other diploid anuran genomes to which we compared *Sp. multiplicata*, which range from 21,067 to 25,846 genes (Hellsten *et al.* 2010; Hammond *et al.* 2017; Edwards *et al.* 2018) (Supplementary Table 5; Supplementary Figure 2).

### Genes with elevated copy numbers in Sp. multiplicata

**Figure 2:**
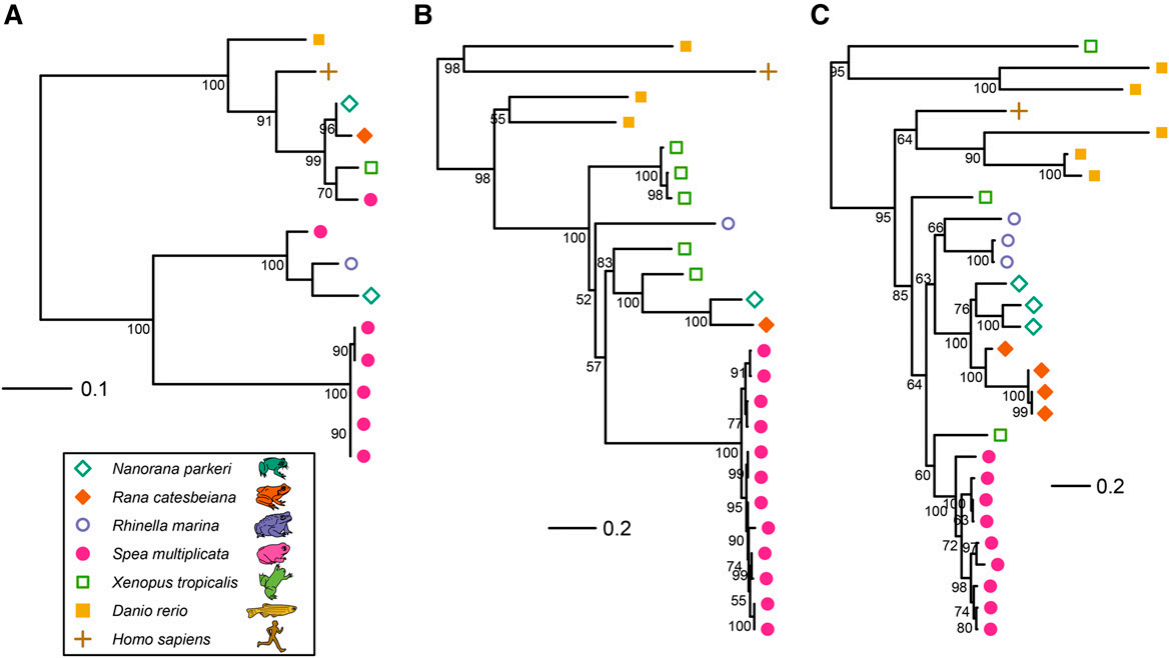
Gene trees for genes with elevated copy numbers in *Sp. multiplicata*. We utilized Usearch (Edgar 2010) to identify gene enrichments in *Spea* compared to four other anurans. We then retrieved copies of the gene in question for humans and zebrafish from Uniprot. Trees were built from alignments including only sites where all species had data. Distance is in number of substitutions per site. Node labels are shown for all nodes with at least 50% bootstrapping support over 100 iterations. A *hyaluronan synthase 3* (*hyas3*; seven copies of *Sp. multiplicata*); B *nodal* (12 copies); and C *zona pellucida glycoprotein 3* (*zp3*; nine copies).

We examined the gene content of *Sp. multiplicata* in greater detail. At a False Discovery Rate (FDR) of 0.05, Gene Ontology (GO) enrichment analysis failed to identify any significant differences relative to the other diploid anuran genomes with available annotations (Methods). However, comparison of these genomes revealed that three specific gene families were expanded in *Sp. multiplicata* (Supplementary Table 6; Methods). These were *hyaluronan synthase* (*hyas*), *nodal* (*nod*), and *zona pellucida glycoprotein* (*zp3*) (Figure 2). Hyaluronan is a component of extracellular matrices, which play an important role in cell adhesion, differentiation, and migration throughout the body (Spicer and Mcdonald 1998). Whereas the other anurans have one to three annotated copies of *hyas*, *Sp. multiplicata* has seven (Figure 2A). As for *nodal*, this gene encodes a cytokine that plays a key role in mesoderm formation and body patterning during embryogenesis and development in deuterostomes (Osada and Wright 1999; Schier and Shen 2000; Takahashi *et al.* 2000). Vertebrates exhibit substantial diversity in *nodal* content: humans have just a single copy of *nodal*, zebrafish has three, and the other sequenced, diploid anurans have nine or fewer (Figure 2) (Takahashi *et al.* 2006). In comparison, we found evidence that *Sp. multiplicata* has at least 12 copies of *nodal* (Figure 2B; Supplementary Table 7). The *nodal* gene family also expanded in *X. tropicalis* (Terai *et al.* 2006; Hellsten *et al.* 2010). When we compared the 12 *Sp. multiplicata* copies of nodal to those present in *X. tropicalis,* we found that all were most similar to *xnr6*. To further investigate this, we relaxed our criteria and included matches to all the nodals in the SWISSprot database. We found evidence for potentially as many as 24 copies of *nodal* in Sp. multiplicata: 22 copies were most similar to *xnr6,* while the remaining two copies were most similar to another gene in *X. tropicalis, xnr2* (Supplementary Table 7; Methods). Unlike most other *nodal* copie*s* in *Xenopus*, *xnr6* acts in a cell-autonomous manner (Takahashi *et al.* 2000) and plays a key role in mesoendoderm specification (Luxardi *et al.* 2010). In contrast, *xnr2* acts later in development and is a mesoderm-inducing factor (Agius *et al.* 2000). Lastly, *zp3* encodes a protein component of sperm-binding glycoproteins in the egg’s zona pellucida (Harris *et al.* 2009). While the four other diploid anuran genomes had four or fewer copies of *zp3*, *Sp. multiplicata* had nine (Figure 2C).

### Factors contributing to anuran genome size differences

**Figure 3:**
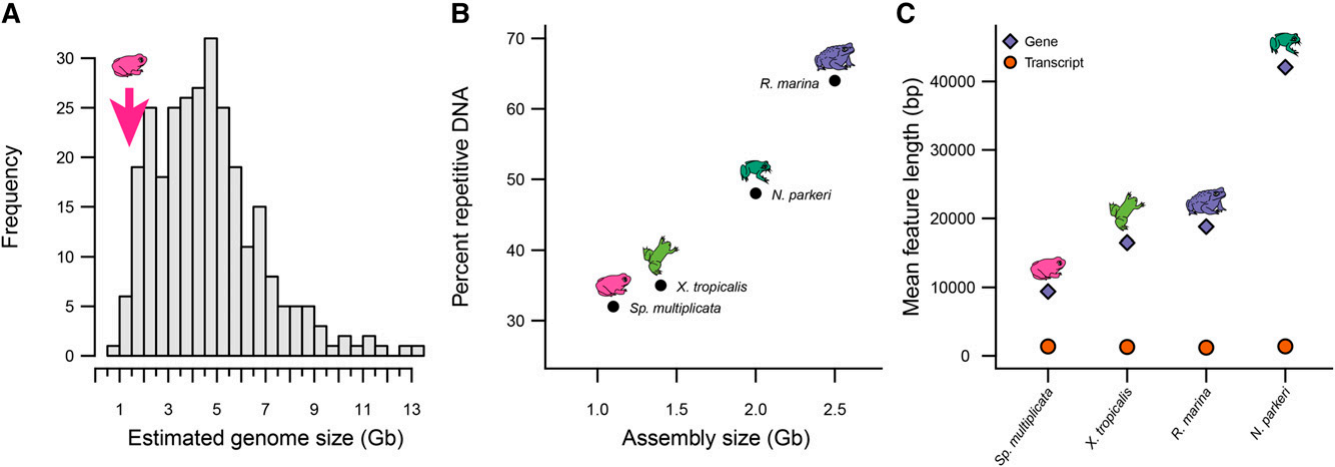
Factors contributing to anuran genome size differences. a Estimated genome sizes (Supplementary Note 1) among 284 species of anurans (Gregory 2018); arrow, estimated genome size of *Spea* from densitometry. b Assembly size and c gene and transcript lengths of *Sp. multiplicata* compared to three other sequenced, diploid anurans.

*Spea multiplicata* has a smaller genome than most anurans (Figure 3A), including the four other sequenced diploid anurans (Supplementary Table 5). We sought to identify genomic features that explain these genome size differences (Methods). In our analyses, we excluded *R. catesbiana* because of its fragmented gene set (Supplementary Note 2), which is a result of its comparatively large and very repetitive genome (Hammond *et al.* 2017). Among the other four assemblies, there is a more than twofold range of genome sizes (Figure 3B; Supplementary Table 5). Repetitive DNA content––*i.e.*, percent of a genome assembly comprised of any class of repetitive DNA––exhibited a near perfect correlation with genome size (*ρ* = 0.99, *P =* 0.01; Figure 3B). Repetitive DNA exhibits an almost twofold range across the four species, suggesting it accounts for most, but not all, of the differences in genome size.

We also found smaller contributors to genome size differences (Supplementary Table 5). Number of annotated genes was strongly correlated with genome size (*ρ* = 1, *P <* 0.0001), although we note that this feature is highly sensitive to assembly and annotation methods. Additionally, gene length, calculated as the number of exonic and intronic bases within a protein-coding gene, varied substantially across the four genomes. *Sp. multiplicata* has appreciably smaller genes (∼9.4 kb on average) than the other species (≥ ∼16.5 kb on average; Figure 3C). These differences in gene size are driven by variability in intronic DNA, as the exonic portions of genes are approximately the same in the four species (∼1.2 to ∼1.3 kb).

### Identification of genes showing evidence of positive selection

Spadefoots, in particular those in the genus *Spea*, exhibit remarkable adaptations, especially for living in desert conditions where most anurans would not survive. We therefore used the *Sp. multiplicata* genome as a resource for studying adaptive evolution across spadefoot species. To do so, we obtained short-read sequencing data for an additional *Spea* species, *Sp. bombifrons*, as well as for two species of the closest sister taxa, *Sc. holbrookii*, and *Sc. couchii* (Methods). Using these data, we generated near complete four-species nucleotide and amino acid alignments for 1,967 single-copy, protein-coding genes (Methods). We then estimated *dN*/*dS* (ω) within each genus and tested whether genes showed evidence of different selection pressures in the two genera (Supplementary Table 8; Methods).

**Figure 4:**
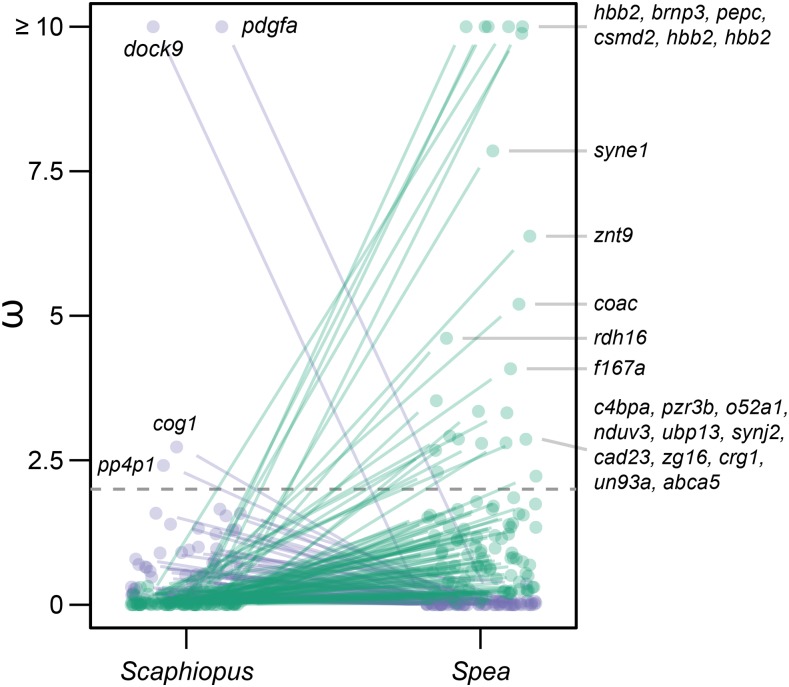
Identification of genes showing evidence of positive selection in two genera of spadefoots. ω values for genes showing significantly different selection between sequenced *Spea* and *Scaphiopus* species. Genes with a ω >= 2 (horizontal dashed line) were considered as putatively under positive selection in this study. We found more genes (*n* = 22) that met these criteria in *Spea* than in *Scaphiopus* (*n* = 4). Significant genes are labeled by their closest match in the SWISSprot database.

At an FDR of 0.05, 172 genes had significantly different ω values between *Spea* and *Scaphiopus* (Figure 4, Methods). Of these, 26 genes (22 in *Spea* and 4 in *Scaphiopus*) exhibited evidence of positive selection in one of the genera, here operationally defined as ω > 2 (Methods). In every case, genes under positive selection in one genus were under purifying selection in the other. The remaining genes either showed evidence of neutral evolution in one genus but not the other, or exhibited differing degrees of purifying selection between the genera (Figure 4).

We determined the functions of the 22 genes that were under positive selection in *Spea* (Methods). These genes played roles in eye function, immune function, metabolism and digestion, oxygen transport, and smell (Supplementary Table 9). Gene ontology (GO) analysis of these genes identified 13 biological processes that were enriched (Supplementary Table 10), including: coenzyme biosynthesis, immune function, intracellular organization, lipid metabolism and transport, photoreceptor cell maintenance, and zinc ion transport.

### Insights into adaptive hybridization from the transcriptome

Finally, we leveraged the *Sp. multiplicata* genome to gain insights into the genomic factors that might contribute to adaptive hybridization that is observed between the *Spea* species, *Sp. multiplicata* and *Sp. bombifrons* (Pfennig and Simovich 2002; Pfennig 2007; Pierce *et al.* 2017). Specifically, we first performed 3′ RNA-seq on seven *Sp. bombifrons* and seven *Sp. multiplicata* tadpoles (Methods). The tadpoles had different parents that were sampled from distinct geographic locations, allowing us to identify those genes that exhibit fixed expression differences between the two species.

In total, we obtained measurements in all 14 tadpoles for 10,695 annotated genes (Supplementary Data 2; Methods). At an FDR of 0.05, we identified 5,865 genes (54.8% of all genes) that were differentially expressed between the species (Supplementary Data 3; Methods). Among these genes, 53.3% exhibited higher expression in *Sp. bombifrons* and 46.7% showed higher expression in *Sp. multiplicata*. On average, differentially expressed genes had a 3.8-fold difference in transcript levels between the two species. However, differences as small as 1.twofold and as large as 117.7-fold were detected. Notably, many genes show sizable changes in transcription between the species; for example, 10% of all identified genes exhibited an at least 6.5-fold expression difference.

**Figure 5:**
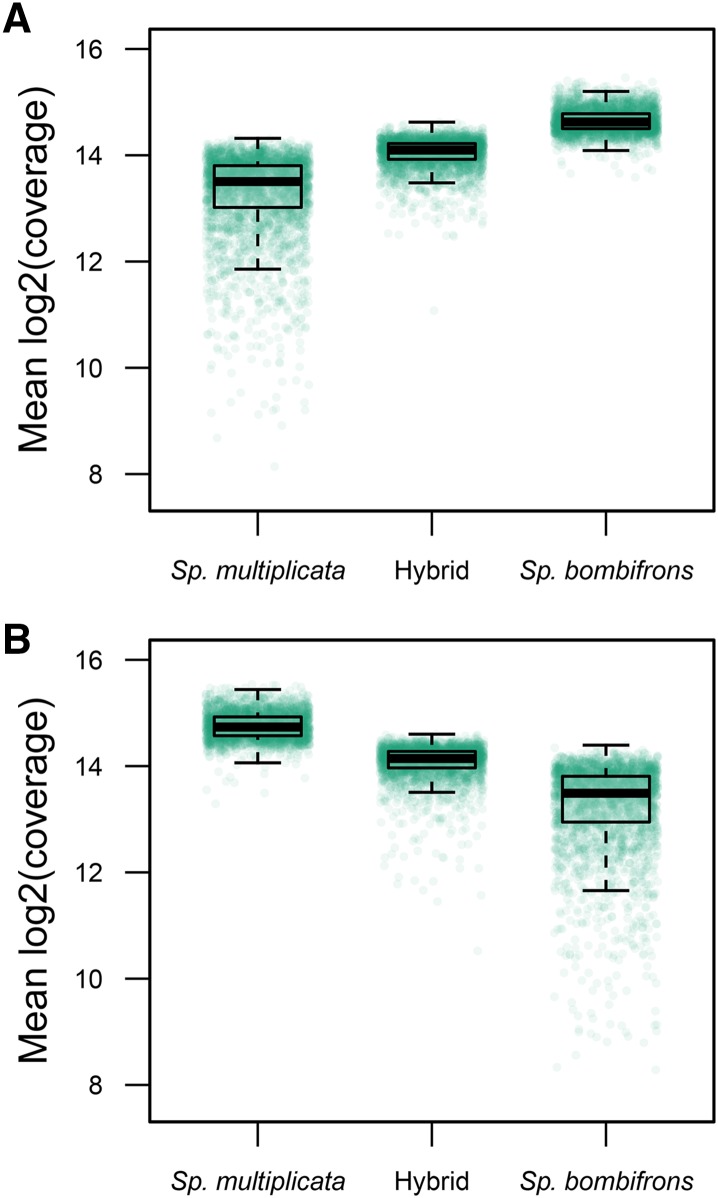
Gene expression analysis in *Spea* hybrids. Genes exhibiting differential expression between *Sp. bombifrons* and *Sp. multiplicata* are shown in these pure species and their hybrids. Genes expressed at a higher level in *Sp. bombifrons* (n = 3,123) are shown in A, while genes expressed at a higher level in *Sp. multiplicata* (n = 2,742) are shown in B. Each point represents the average expression level of a single gene across all samples in a given class.

We also used 3′ RNA-seq to measure the expression of the same 10,695 genes in 14 F_1_ hybrid tadpoles produced by mating *Sp. bombifrons* and *Sp. multiplicata* adults obtained from multiple geographic locations (Supplementary Data 3; Methods). For 93.8% of all genes differentially expressed between the species, transcript levels were higher in the hybrid tadpoles than in the lower expressing parent-species tadpoles (Supplementary Data 3). When we focused on the 586 genes showing greater than 6.5-fold expression differences between the species, this proportion was even higher: 585 of 586 (99.8%) had transcript levels in the hybrid tadpoles that were above the lower expressing parent-species tadpoles. Indeed, hybrids exhibited expression levels close to the average of their parents (Figure 5).

## Discussion

The *Sp. multiplicata* genome advances research not only in New World spadefoot toads, but also in anurans more generally. Anurans are noted for their genome size variation, so they are powerful models for evaluating how and why genome size evolves (Liedtke *et al.* 2018; Mueller and Jockusch 2018). Recent findings indicate that anurans’ continuous rate of genome size evolution is higher on average than other amphibian clades, and that life history—specifically larval development time––is positively correlated with genome size (Liedtke *et al.* 2018). The small genome size and rapid development of *Sp. multiplicata* exemplify this relationship.

The genomic factors that contribute to variation in genome size remain an issue of active inquiry (Petrov 2001; Gregory 2005; Lynch 2007). We found that, despite their smaller genome, spadefoots are similar to other sequenced anurans in terms of number and type of genes. Thus, *Spea*’s smaller genome appears to derive from diminished repetitive and intronic DNA, which is consistent with the prevailing hypothesis that genome size has undergone gradual change––as opposed to abrupt change––throughout much of amphibian evolutionary history (Chrtek *et al.* 2009; Liedtke *et al.* 2018). As more amphibian genomes become available, greater insights will be attained into the evolutionary and genomic factors that contribute to genome size evolution in this clade.

Because of their diverse adaptations (Duellman and Trueb 1986; Halliday 2016), anurans are also classic models in ecology, evolution, and development. The *Sp. multiplicata* genome will help provide additional insights into these fields. For example, we found that spadefoots possess modest increases in copies of genes involved in development and fertilization, most notably in the key developmental regulator *nodal*. Studies using *X. tropicalis* have shown that *nodal* paralogs exhibit different spatiotemporal expression patterns during development, which play roles in the formation of distinct tissues (Osada and Wright 1999; Charney *et al.* 2017). The numerous copies of *nodal* in *Sp. multiplicata* might contribute to this species’ remarkable phenotypic plasticity by assigning specialized functions to different copies during development and/or facilitating rapid bursts of transcription following abrupt changes in the environment (*e.g.*, diet or pond volume) that allows alternative traits to develop quickly. Although it is unlikely to be the sole contributing factor, such gene proliferation might help explain how key developmental pathways become environmentally sensitive without disrupting overall organism form and function (West-Eberhard 2003). The *Sp*. *multiplicata* genome, along with other anuran genomes, will enable future work that can address this and related issues in evolution and development.

As an example of how the *Sp. multiplicata* genome can facilitate new lines of genomic research in this system, we report a large-scale scan for genes showing evidence for selection in *Spea* and/or *Scaphiopus*. Both genera include desert-adapted and extremely rapid developing species, but *Scaphiopus* cannot produce carnivore-morph tadpoles (Figure 1E). We identified 26 genes (22 in *Spea* and 4 in *Scaphiopus*) exhibiting signatures of potential positive selection (Figure 4). Interestingly, genes under positive selection in one genus were exclusively under purifying selection in the other genus. This suggests that the two genera, while ecologically similar in many ways, are nevertheless experiencing, and responding to, distinct selection pressures. A key aspect of spadefoot biology that could be impacted by these putatively selected genes in *Spea* is the production of carnivores, which frequently feed on other tadpoles. Consumption of other tadpoles increases risk of pathogen transmission (Pfennig *et al.* 1998) and might thereby drive the observed positive selection on the immune function genes in *Spea*. The *Sp. multiplicata* genome will enable explicit testing of these hypotheses and allow for deeper investigation of the mechanisms underlying both adaptive evolution and the diversification of phenotypes among species that share the same environments.

Further, we show how the *Sp. multiplicata* genome enables genomic research on the evolution of hybridization. Here, we analyzed gene expression differences between *Sp. multiplicata* and *Sp. bombifrons*, focusing on same-age tadpoles reared in a controlled environment. We found evidence that more than half of the genes in the genome exhibit differential expression between these species (Figure 5), which are the most distantly related in their genus (Wiens and Titus 1991; Zeng *et al.* 2014). The number of genes showing higher expression in *Sp. multiplicata vs. Sp. bombifrons* was roughly equal, which is consistent with the notion that species differences accumulate via genetic drift. Yet, despite these genome-wide expression differences, *Sp. multiplicata* and *Sp. bombifrons* interbreed and produce viable hybrid offspring (Pfennig and Simovich 2002; Pfennig *et al.* 2012). Although we cannot rule out gene expression differences arising due to variability in developmental timing, F_1_ hybrid gene expression being intermediate at those genes that differ in expression between *Sp. multiplicata* and *Sp. bombifrons* may play a role in adaptive hybridization in *Spea* (Pfennig 2007). Given that hybridization’s role in the origin and distribution of species remains a topic of keen interest (Abbott *et al.* 2013; Pfennig *et al.* 2016), the *Sp. multiplicata* genome provides a new resource for evaluating how genomic factors and ecological context interact to determine how and when hybridization is adaptive.

In summary, spadefoots possess many striking ecological, evolutionary, and developmental features that are now possible to study at the genomic level. Moving forward, the genome described in this paper should provide a critical foundation for analyzing this substantial diversity within and between spadefoot species, as well as for more deeply understanding the mechanisms producing the features that distinguish spadefoots from other anurans.
